# Effects of Co-Culture Media on Hepatic Differentiation of hiPSC with or without HUVEC Co-Culture

**DOI:** 10.3390/ijms18081724

**Published:** 2017-08-07

**Authors:** Nora Freyer, Selina Greuel, Fanny Knöspel, Nadja Strahl, Leila Amini, Frank Jacobs, Mario Monshouwer, Katrin Zeilinger

**Affiliations:** 1 Berlin-Brandenburg Center for Regenerative Therapies (BCRT), Charité—Universitätsmedizin Berlin, Campus Virchow-Klinikum, 13353 Berlin, Germany; selina.greuel@charite.de (S.G.); fanny.knoespel@gmx.de (F.K.); nadja.strahl@outlook.com (N.S.); leila.amini@charite.de (L.A.); katrin.zeilinger@charite.de (K.Z.); 2 Janssen Research and Development, 2340 Beerse, Belgium; fjacobs1@its.jnj.com (F.J.); mmonshou@its.jnj.com (M.M.)

**Keywords:** human induced pluripotent stem cells (hiPSC), hepatic differentiation, human umbilical vein endothelial cells (HUVEC), co-culture

## Abstract

The derivation of hepatocytes from human induced pluripotent stem cells (hiPSC) is of great interest for applications in pharmacological research. However, full maturation of hiPSC-derived hepatocytes has not yet been achieved in vitro. To improve hepatic differentiation, co-cultivation of hiPSC with human umbilical vein endothelial cells (HUVEC) during hepatic differentiation was investigated in this study. In the first step, different culture media variations based on hepatocyte culture medium (HCM) were tested in HUVEC mono-cultures to establish a suitable culture medium for co-culture experiments. Based on the results, two media variants were selected to differentiate hiPSC-derived definitive endodermal (DE) cells into mature hepatocytes with or without HUVEC addition. DE cells differentiated in mono-cultures in the presence of those media variants showed a significant increase (*p* < 0.05) in secretion of α-fetoprotein and in activities of cytochrome P450 (CYP) isoenzymes CYP2B6 and CYP3A4 as compared with cells differentiated in unmodified HCM used as control. Co-cultivation with HUVEC did not further improve the differentiation outcome. Thus, it can be concluded that the effect of the used medium outweighed the effect of HUVEC co-culture, emphasizing the importance of the culture medium composition for hiPSC differentiation.

## 1. Introduction

Human induced pluripotent stem cells (hiPSC) hold great promise for application in cell therapies [1], but also in disease research [1,2] and drug toxicity testing with in vitro models [3]. In pharmacological research, there is a particular need for hepatic cells due to the central role of the liver in drug metabolism and toxicity [4,5]. Previous in vitro studies on hepatic drug toxicity using hepatocyte-like cells (HLC) differentiated from hiPSC showed promising results [6,7]. Despite these encouraging outcomes, the efficiency of the differentiation process of hiPSC towards functional HLC for further use, including hepatotoxicity assessment, remains low [8]. Several research groups managed to generate hiPSC-derived HLC with 60% [9] up to 80–85% [10] of differentiated cells being characterized by the expression of several hepatic markers, including albumin. However, hiPSC-derived HLC showed only 10% of the urea and albumin production capacity compared with primary human hepatocytes [9] and cytochrome P450 (CYP) isoenzyme activities of the generated HLC were almost 30-fold lower than those of primary human hepatocytes [9]. Baxter et al. characterized human embryonic stem-cell derived HLC as being similar to fetal rather than to adult hepatocytes in terms of their metabolic profile [11]. Therefore, further improvement of the differentiation and maturation process is needed to generate fully functional HLC for clinical or in vitro use.

Current protocols for differentiation of hiPSC towards HLC mostly use a variation of a three-step differentiation protocol including differentiation into definitive endoderm (DE) cells, generation of hepatoblasts and further maturation into HLC [12]. DE differentiation is generally induced by the use of activin A [13,14] and Wnt3a [13,15]. Differentiation into hepatoblasts is supported by the addition of fibroblast growth factors (FGF) and bone morphogenetic proteins (BMP) [16,17] and hepatocyte maturation is induced by using hepatocyte growth factor (HGF) as well as oncostatin M (OSM) [18]. In addition to the optimization of the differentiation media and supplements, overexpression of hepatic transcription factors such as hepatocyte nuclear factor 4 α (HNF4A) [19] and manipulation of miRNA expression [20,21] were investigated aiming to improve hepatic maturation of hiPSC. Further support of the maturation of gained HLC was achieved by transferring the differentiation process to a 3D-culture system [6,22,23].

A promising approach to enhance the differentiation outcome and hepatic functionality of hiPSC-derived HLC can be seen in applying co-cultures with non-parenchymal liver cells. Matsumoto et al. observed that endothelial cells are essential for early organ development prior to the formation of a functioning local vasculature [24]. Takebe and colleagues recapitulated the early liver development by cultivating hiPSC-derived endodermal cells with human umbilical vein endothelial cells (HUVEC) and human mesenchymal stem cells. The observed expression profiles in the resulting hiPSC-liver buds were closer to native human liver tissue than hiPSC-derived HLC differentiated without HUVEC co-culture [25]. Since the cell behavior within such co-cultures relies on specific media compositions, special attention has to be given to the co-culture medium used to provide suitable conditions for all included cell types.

**Figure 1:**
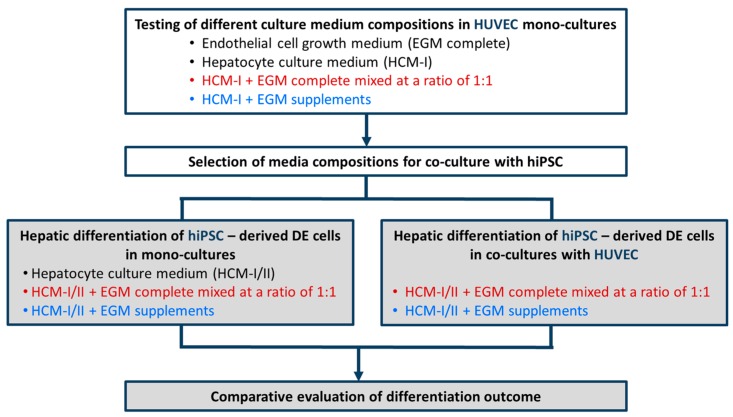
Schematic outline of experimental procedures for media testing in mono-cultures of human umbilical vein endothelial cells (HUVEC, white boxes) and for hepatic differentiation of hiPSC-derived definitive endodermal (DE) cells with or without HUVEC (grey boxes). Different mixtures of hepatocyte culture medium (HCM) and endothelial cell growth medium (EGM) were tested. Colors used for the different media compositions correspond to those used in the graphs.

Within the scope to develop a co-culture model consisting of hiPSC-derived DE cells and HUVEC, a two-step approach was applied as shown in Figure 1. First, culture media used for endothelial cells or hepatocytes as well as mixtures thereof were tested in HUVEC mono-cultures. Based on the results, two of those media were selected to differentiate hiPSC-derived DE cells into mature hepatocytes in the presence or absence of HUVEC. Hepatic differentiation of hiPSC in mono- or co-cultures was assessed by measuring the protein and gene expression of stage-specific markers as well as the functionality of pharmacological relevant CYP isoenzymes.

## 2. Results

As a prerequisite for co-culture, a culture medium permitting endothelial cell maintenance during differentiation of hiPSC-derived DE cells towards the hepatic fate had to be determined. Therefore, HUVEC were cultured in the presence of 100% endothelial cell growth medium, consisting of basal medium and supplements (EGM complete), 100% hepatocyte culture medium (HCM-I), HCM-I and EGM complete mixed at a ratio of 1:1 (HCM-I + EGM complete) or HCM-I enriched with endothelial cell growth supplements (HCM-I + EGM supplements).

### 2.1. Effect of Culture Media Variations on Mono-Cultures of Human Umbilical Vein Endothelial Cells (HUVEC)

**Figure 2:**
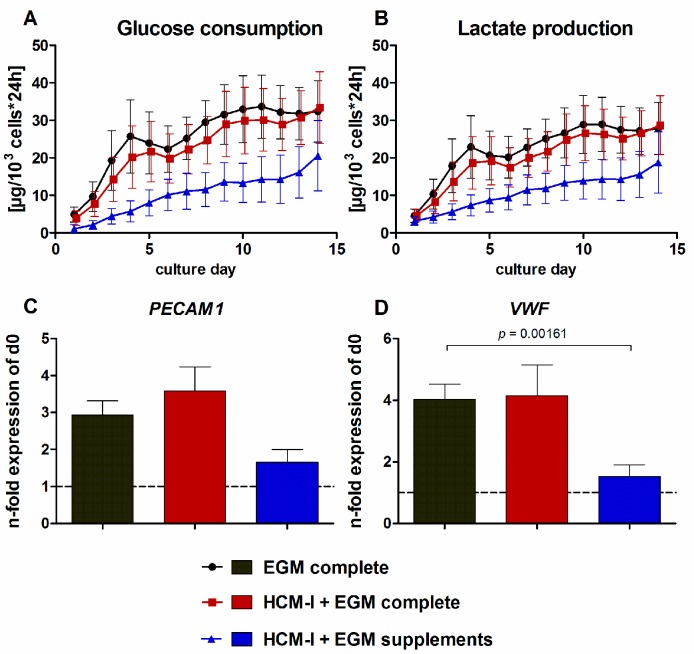
Effect of different media compositions on mono-cultures of human umbilical vein endothelial cells (HUVEC). The cells were cultured over 14 days in endothelial cell growth medium, consisting of basal medium and supplements (EGM complete), in a 1:1 mixture of hepatocyte culture medium and EGM complete (HCM-I + EGM complete) or in HCM enriched with endothelial cell growth supplements (HCM-I + EGM supplements). The graphs show time-courses of glucose consumption (**A**) and lactate production (**B**) as well as gene expression analyses of the endothelial cell markers platelet endothelial cell adhesion molecule 1 (*PECAM1*) (**C)** and von Willebrand factor (*VWF*) (**D**). Fold changes of mRNA expression were calculated relative to HUVEC before starting the experiments (d0) with normalization to glyceraldehyde-3-phosphate dehydrogenase (*GAPDH*) expression by the ∆∆Ct method. The area under the curve was calculated for time-courses of biochemical parameters and differences between groups were detected using the unpaired, two-tailed Student’s *t*-test; for glucose consumption and lactate production *n* = 6, gene expression analysis *n* = 3, mean ± standard error of the mean.

The use of pure HCM-I for HUVEC cultivation resulted in rapid cell disintegration and detachment and was therefore not further evaluated (data not shown). The results from HUVEC cultures treated with HCM-I + EGM complete, with HCM-I + EGM supplements or with EGM complete as control are shown in Figure 2. The curve progressions displaying glucose consumption rates of HUVEC cultivated in EGM complete or HCM-I + EGM complete were similar, showing an increase until day 4 and a slight decrease until day 6 followed by a slow, but stable increase until day 14 (Figure 2A). Values were only marginally lower in HCM-I + EGM complete than in EGM complete. In contrast, HUVEC grown in HCM-I + EGM supplements showed considerably lower glucose consumption rates over the whole culture period (Figure 2A). Values of lactate production mirrored those of glucose consumption (Figure 2B). Release of lactate dehydrogenase (LDH) as a marker for cell damage was detected at basal levels of maximally 3 U/L for all conditions (data not shown). Gene expression analysis of the endothelial cell markers platelet and endothelial cell adhesion molecule 1 (*PECAM1*) and von Willebrand factor (*VWF*) revealed a 3- to 3.5-fold increase in *PECAM1* gene expression and a 4-fold increase in *VWF* gene expression when using EGM complete or HCM-I + EGM complete (Figure 2C,D). In contrast, the expression levels of both, *PECAM1* and *VWF*, remained stable in HUVEC cultured in HCM-I + EGM supplements throughout the culture period of 14 days.

**Figure 3:**
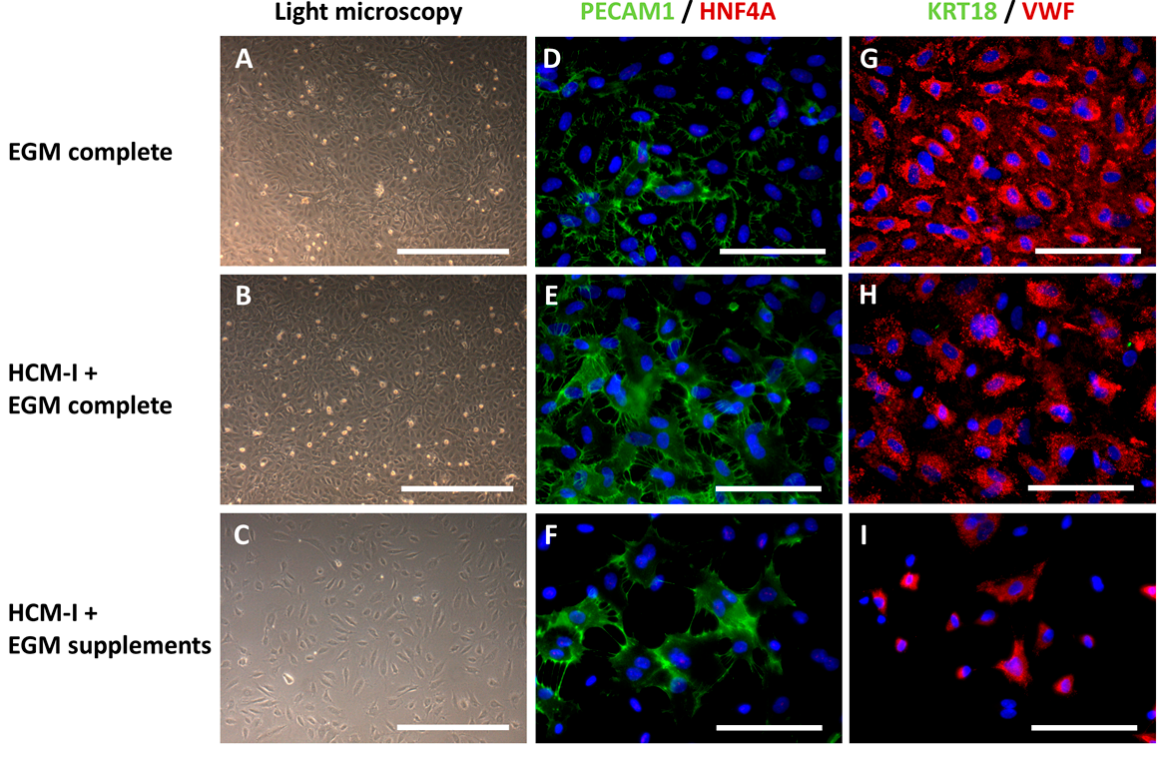
Light microscopy and immunocytochemical staining of mono-cultures of human umbilical vein endothelial cells (HUVEC) after cultivation over 14 days in endothelial cell growth medium, consisting of basal medium and supplements (EGM complete), hepatocyte culture medium and EGM complete mixed at a ratio of 1:1 (HCM-I + EGM complete) or HCM enriched with endothelial cell growth supplements (HCM-I + EGM supplements). The pictures show light microscopic photographs (**A–C**), staining of the endothelial cell marker platelet endothelial cell adhesion molecule 1 (PECAM1) and the hepatocyte marker hepatocyte nuclear factor 4 α (HNF4A) (**D–F**), staining of the hepatocyte marker cytokeratin 18 (KRT18) and the endothelial cell marker von Willebrand factor (VWF) (**G–I**). Nuclei were counter-stained with Dapi (blue). Scale bars correspond to 500 µm for light microscopy and to 100 µm for immunofluorescence.

In accordance to the results of glucose and lactate measurement, cultures grown in EGM complete showed the highest cell density (Figure 3A), followed by those cultured in HCM-I + EGM complete (Figure 3B). Cultivation in HCM-I + EGM supplements resulted in a distinctly lower cell density as compared with the other media investigated (Figure 3C). Immunocytochemical staining of endothelial and hepatocyte markers was performed in HUVEC mono-cultures to evaluate the influence of the tested media on protein expression (Figure 3). Relative percentages of stained cells are provided in Table S1. In all conditions, the two endothelial cell markers PECAM1 and VWF could clearly be observed, whereas the two hepatocyte markers HNF4A and cytokeratin 18 (KRT18) were not detectable (Figure 3D–I). However, the proportion of cells expressing endothelial cell markers differed between the conditions. Almost all cells (91 ± 6%) cultured in EGM complete were positive for PECAM1 (Figure 3D), whereas only around 70% of the cells cultured in HCM-I + EGM complete or in HCM-I + EGM supplements showed PECAM1 immunoreactivity (Figure 3E,F). Immunoreactivity for VWF was observed in approximately 80% of the cells when grown in EGM complete or in HCM-I + EGM complete (Figure 3G,H), while only 62 ± 12% of the cells cultured in HCM-I + EGM supplements appeared positive for this marker (Figure 3I).

### 2.2. Hepatic Differentiation of hiPSC-Derived Definitive Endoderm (DE) Cells with or without HUVEC Co-Cultivation Using Different Co-Culture Media

As maintenance and proliferation of endothelial cells might have positive effects on hepatic differentiation of hiPSC, both, HCM-I/II + EGM complete and HCM-I/II + EGM supplements, were tested for their suitability to induce hepatic differentiation in hiPSC-derived DE cells maintained in mono-culture or in co-culture with HUVEC. The results were compared with pure HCM-I/II, used as a positive control. HCM-II is based on HCM-I, but is further supplemented with OSM for the last four days of differentiation.

#### 2.2.1. Morphological Characteristics of hiPSC-Derived Hepatocyte-like Cells (HLC) and Co-Cultured HUVEC

**Figure 4:**
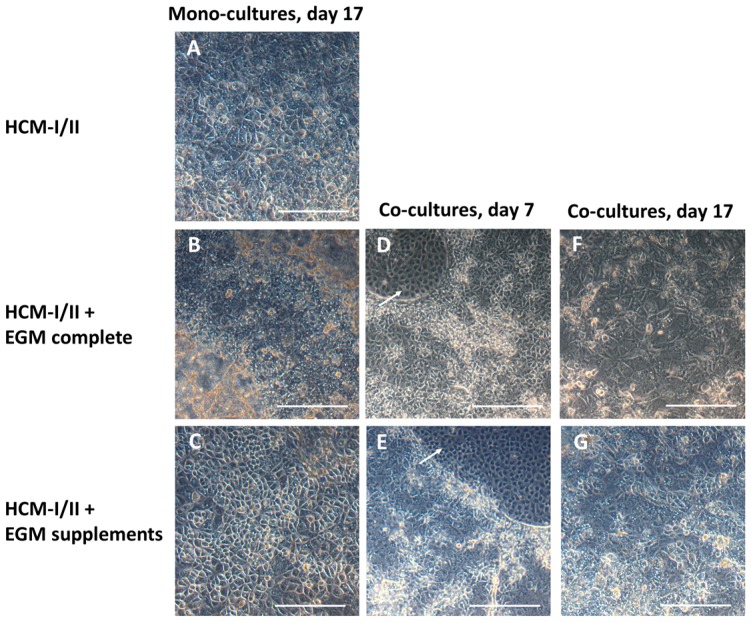
Morphology of human induced pluripotent stem cells (hiPSC) after hepatic differentiation over 17 days in different media and/or in co-culture with human umbilical vein endothelial cells (HUVEC). The pictures show hiPSC after hepatic differentiation in mono-culture over 17 days in hepatocyte culture medium (HCM-I/II) (**A**), in a 1:1 mixture of hepatocyte culture medium and endothelial cell growth medium EGM, consisting of basal medium and supplements (HCM-I/II + EGM complete) (**B**) or in HCM enriched with EGM supplements (HCM-I/II + EGM supplements) (**C**); hiPSC after hepatic differentiation in co-culture with HUVEC on day 7 of differentiation using HCM-I/II + EGM complete (**D**) or HCM-I/II + EGM supplements (**E**); hiPSC after hepatic differentiation in co-culture with HUVEC on day 17 of differentiation using HCM-I/II + EGM complete (**F**) or HCM-I/II + EGM supplements (**G**). HUVEC were growing in free spaces between the hiPSC (arrows). Scale bars correspond to 300 µm.

Light microscopic investigation at the end of hepatic differentiation showed that hiPSC-derived HLC maintained without HUVEC co-culture displayed a polygonal shape typical for hepatocytes in all tested media compositions (Figure 4A–C). Use of HCM-I/II + EGM complete resulted in a rather heterogeneous morphology showing large areas of overgrowth (Figure 4B), while HLC differentiated in HCM-I/II or HCM-I/II + EGM supplements appeared more homogeneous (Figure 4A,C). In co-culture experiments, using HCM-I/II + EGM complete or HCM-I/II + EGM supplements as culture media, the HUVEC grew in distinct areas between the hiPSC-derived DE cells until day 7 of the differentiation process (Figure 4D,E). Afterwards, HUVEC progressively infiltrated the hiPSC clusters and at the end of hepatic differentiation (day 17) they could hardly be discriminated from HLC (Figure 4F,G). In both medium conditions, HLC co-cultivated with HUVEC were less homogeneous in their morphology and culture behavior (Figure 4F,G) as compared with the corresponding hiPSC mono-cultures (Figure 4B,C).

#### 2.2.2. Gene Expression of Stage-Specific and Endothelial Cell Markers

**Figure 5:**
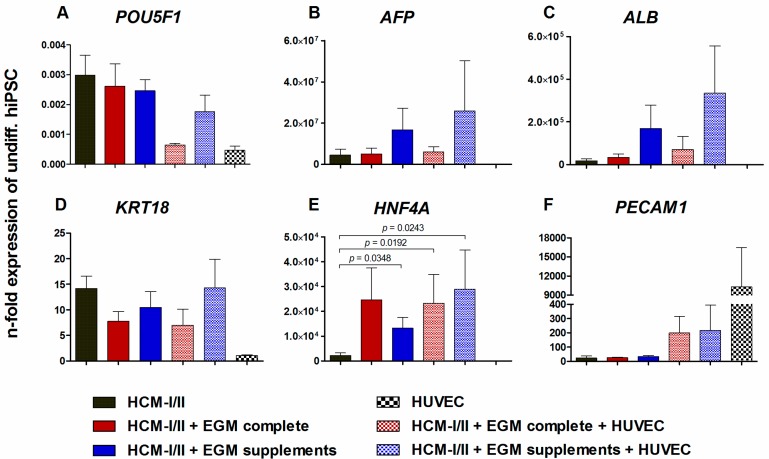
Effect of different media compositions and/or co-culture with human umbilical vein endothelial cells (HUVEC) on mRNA expression of stage-specific markers after hepatic differentiation of human induced pluripotent stem cells (hiPSC). Differentiation of definitive endodermal cells was performed over 14 days using hepatocyte culture medium (HCM-I/II), a 1:1 mixture of hepatocyte culture medium and endothelial cell growth medium, consisting of basal medium and supplements (HCM-I/II + EGM complete) or HCM enriched with endothelial cell growth supplements (HCM-I/II + EGM supplements) with or without HUVEC addition. In addition mRNA expression analysis was performed with HUVEC mono-cultures cultured in EGM complete as control. Graphs show POU class 5 homeobox 1 (*POU5F1*, **A**), α-fetoprotein (*AFP*, **B**), albumin (*ALB*, **C**), cytokeratin 18 (*KRT18*, **D**), hepatocyte nuclear factor 4 α (*HNF4A*, **E**) and platelet endothelial cell adhesion molecule 1 (*PECAM1*, **F**). Fold changes of mRNA expression were calculated relative to undifferentiated hiPSC with normalization to glyceraldehyde-3-phosphate‎ dehydrogenase (*GAPDH*) expression by the ∆∆*C*t method. Differences between HCM and all other groups and differences between test media and their corresponding co-cultures were detected with the unpaired, two-tailed Student’s *t*-test, *n* = 8; Co-cultures: *n* = 3; mean ± standard error of the mean.

To evaluate the state of differentiation, mRNA expression of stage specific markers in HLC was analyzed relative to undifferentiated hiPSC. HUVEC cultured in EGM complete were used as a positive control for endothelial cell markers (Figure 5). The expression of the pluripotency gene POU domain, class 5, transcription factor 1 (*POU5F1*, Figure 5A) fell to less than 1% relative to undifferentiated hiPSC in all investigated conditions and was lowest in HUVEC mono-cultures. The fetal hepatocyte marker α-fetoprotein (*AFP*) had distinctly increased in all cultures except for the HUVEC. In particular, cultures differentiated in HCM-I/II + EGM supplements showed a distinct up-regulation of *AFP*, amounting to more than 10^7^-fold in both, HLC mono-cultures or co-cultures with HUVEC (Figure 5B). A similar expression pattern was observed for albumin (*ALB*) as a marker for mature hepatocytes, with a more than 10^5^-fold increase in the cultures differentiated in HCM-I/II + EGM supplements with or without HUVEC co-culture (Figure 5C). However, due to large variances in *AFP* and *ALB* expression, the differences between the investigated conditions were not significant (Figure 5B,C). As additional markers for mature hepatocytes, *KRT18* as well as *HNF4A* were investigated (Figure 5D,E). The expression levels of *KRT18* increased by around 10-fold for all tested conditions except for HUVEC mono-cultures which showed a comparable *KRT18* expression as undifferentiated hiPSC. A more pronounced increase by more than 10^4^-fold was observed for *HNF4A* gene expression in the presence of the different test media, which was significantly higher as compared with pure HCM-I/II (*p* < 0.05; Figure 5E). The expression of the endothelial cell marker *PECAM1* was minimally induced in HLC mono-cultures, whereas a more than 200-fold increase in expression was observed in HLC co-cultured with HUVEC, and HUVEC mono-cultures showed a more than 10^4^-fold higher *PECAM1* expression than undifferentiated hiPSC (Figure 5F).

#### 2.2.3. Immunocytochemical Analysis of Stage-Specific and Endothelial Cell Markers

**Figure 6:**
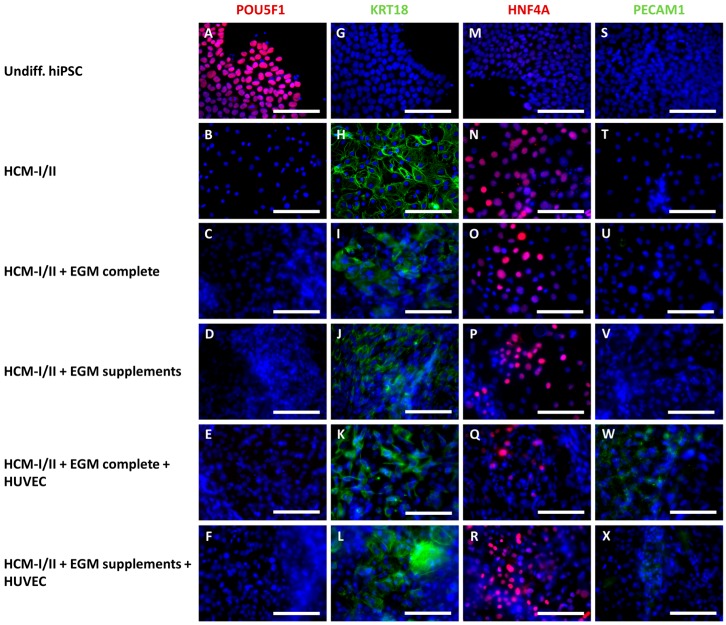
Immunocytochemical staining of human induced pluripotent stem cells (hiPSC) after hepatic differentiation in different media compositions and/or in co-culture with human umbilical vein endothelial cells (HUVEC). Differentiation of definitive endodermal cells was performed over 14 days using hepatocyte culture medium (HCM-I/II), a 1:1 mixture of hepatocyte culture medium and endothelial cell growth medium, consisting of basal medium and supplements (HCM-I/II + EGM complete) or HCM enriched with endothelial cell growth supplements (HCM-I/II + EGM supplements) with or without HUVEC addition. The pictures show the pluripotency marker POU class 5 homeobox 1 (POU5F1, **A–F**); the hepatocyte markers cytokeratin 18 (KRT18, **G–L**) and hepatocyte nuclear factor 4 α (HNF4A, **M–R**) and the endothelial cell marker platelet endothelial cell adhesion molecule 1 (PECAM1, **S–X**). Nuclei were counter-stained with Dapi (blue). Scale bars correspond to 100 µm.

In order to confirm the results of the mRNA analysis, the protein expression of corresponding stage-specific markers in hiPSC-derived HLC was analyzed using immunocytochemical staining (Figure 6). Relative percentages of stained cells are provided in Table S2. In undifferentiated hiPSC cultures, almost all cells (99 ± 3%) were positive for the pluripotency marker POU5F1 (Figure 6A), whereas markers of differentiation (KRT18, HNF4A, PECAM1) were not detectable (Figure 6G,M,S). In contrast, the differentiated cultures showed no immunoreactivity for POU5F1 (Figure 6B–F). The hepatocyte marker KRT18 was clearly expressed in all differentiated cultures (Figure 6H–L). However, the percentage of KRT18 positive cells was 80 ± 6% in cultures incubated with pure HCM-I/II (Figure 6H), whereas in the other experimental groups the proportion of stained cells was distinctly lower, amounting to 60 ± 17% in HCM-I/II + EGM complete + HUVEC (Figure 6K) and less than 50% in the other groups resulting in a heterogeneous appearance (Figure 6I,J,L). Expression of the hepatocyte marker HNF4A was observed in all differentiated cell cultures with the highest percentage of positive cells (60 ± 30%) in HCM-I/II cultures (Figure 6N), followed by HCM-I/II + EGM complete cultures with 28 ± 19% (Figure 6O). All other groups showed 20% or less HNF4A positive cells (Figure 6P–R). The endothelial cell marker PECAM1 was only expressed in HLC cultures differentiated in co-culture with HUVEC (Figure 6W–X), showing a percentage of more than 20% positive cells, while mono-cultures of hiPSC were devoid of PECAM1 (Figure 6S–V).

#### 2.2.4. Secretion of α-Fetoprotein (AFP), Albumin and Urea

**Figure 7:**
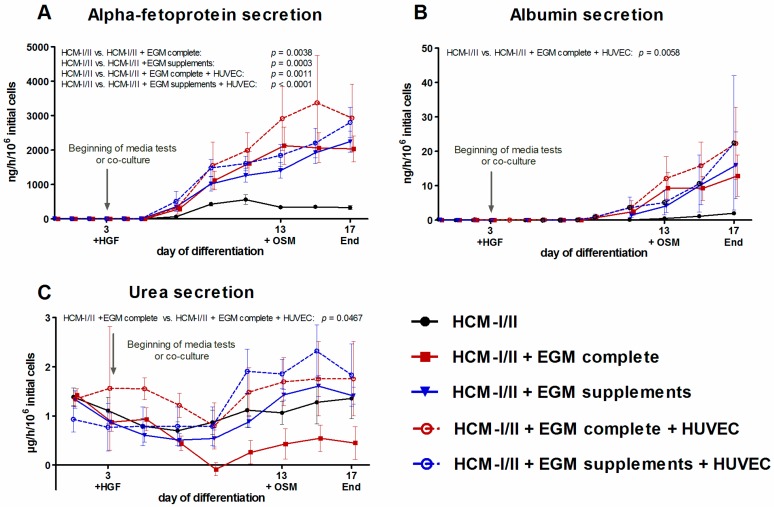
Effect of different media compositions and/or co-culture with human umbilical vein endothelial cells (HUVEC) on secretion of stage-specific markers during differentiation of human induced pluripotent stem cells (hiPSC). Differentiation of definitive endodermal cells was performed over 14 days using hepatocyte culture medium (HCM-I/II), a 1:1 mixture of hepatocyte culture medium and endothelial cell growth medium, consisting of basal medium and supplements (HCM-I/II + EGM complete) or HCM enriched with endothelial cell growth supplements (HCM-I/II + EGM supplements) with or without HUVEC addition. Graphs show the secretion of α-fetoprotein (AFP, **A**), secretion of albumin (**B**) and secretion of urea (**C**). The area under the curve was calculated and differences between HCM and all other groups as well as differences between test media and their corresponding co-cultures were detected using the unpaired, two-tailed Student’s *t*-test, *n* = 8; Co-cultures: *n* = 3; mean ± standard error of the mean.

The capacity of the cells for synthesis of liver-specific proteins was evaluated by measuring the secretion of the fetal albumin precursor protein AFP and of albumin into the culture supernatant (Figure 7). Secretion of AFP was detectable in all culture conditions from differentiation day 7 onwards (Figure 7A). In the HCM-I/II control culture, AFP secretion increased until day 11 reaching a maximum value of 560 ± 137 ng/h/10^6^ initial cells and remained stable afterwards. In contrast, a continuous increase of AFP secretion until the end of the differentiation process on day 17 was observed in both, mono-cultures and co-cultures, treated with HCM-I/II + EGM complete or HCM-I/II + EGM supplements. AFP secretion rates over time, as calculated by the area under the curve, significantly (*p* < 0.05) exceeded the release of this protein in HCM-I/II control cultures, amounting to the 6- to 10-fold on day 17 as compared with HCM-I/II. Mean values of the two co-cultures showed a tendency towards higher rates than the corresponding mono-cultures, though there was no significant difference between both groups. Albumin production was detected in all experimental groups from day 9 onwards (Figure 7B). In HCM-I/II control cultures, secretion rates slowly increased up to 2.0 ± 0.4 ng/h/10^6^ initial cells on day 17, while cultures maintained using the test media clearly showed a steeper increase, attaining 6- to 10-fold higher values as compared with the control. The highest levels of albumin secretion were detected in the co-culture groups with maximal values of 22 ng/h/10^6^ initial cells on day 17. Cells co-cultured with HUVEC in the presence of HCM-I/II + EGM complete produced significantly more albumin than cells in HCM-I/II control cultures (*p* = 0.0058). As a further parameter to assess the functionality of the differentiated cells, urea secretion was measured over time (Figure 7C). Relatively high values were detected at the beginning of differentiation, which decreased until day 9 and then increased again in all experimental groups until day 17. The highest values of urea secretion were detected in co-cultures with HCM-I/II + EGM supplements. Further, urea secretion was significantly increased in the co-culture with HCM-I/II + EGM complete as compared to the corresponding medium control (*p* = 0.0467).

#### 2.2.5. Functional Analysis of Different Cytochrome P450 (CYP) Isoenzymes

**Figure 8:**
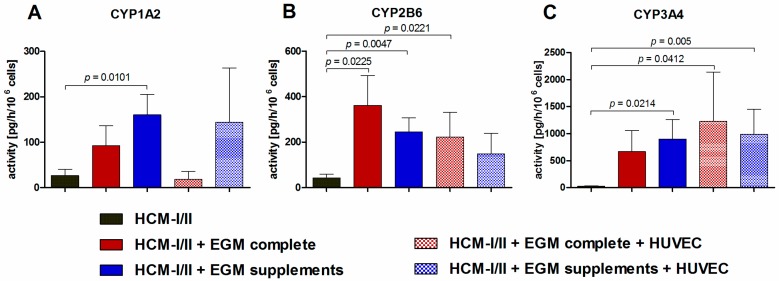
Effect of different media compositions and/or co-culture with human umbilical vein endothelial cells (HUVEC) on activities of cytochrome P450 (CYP) isoenzymes after hepatic differentiation of human induced pluripotent stem cells (hiPSC). Differentiation of definitive endodermal cells was performed over 14 days using hepatocyte culture medium (HCM-I/II), a 1:1 mixture of hepatocyte culture medium and endothelial cell growth medium, consisting of basal medium and supplements (HCM-I/II + EGM complete) or HCM enriched with endothelial cell growth supplements (HCM-I/II + EGM supplements) with or without HUVEC addition. The graphs show activities of CYP1A2 determined by measuring the conversion rates of phenacetin to acetaminophen (**A**), activities of CYP2B6 determined by measuring the conversion rates of bupropion to 4-OH-bupropion (**B**) and activities of CYP3A4 determined by measuring the conversion rates of midazolam to 1-OH-midazolam (**C**) over 6 h. Differences between HCM and all other groups and differences between test media and their corresponding co-cultures were detected using the unpaired, two-tailed Student’s t-test, HCM: *n* = 8; HCM-I/II + EGM complete and HCM-I/II + EGM Supplements: *n* = 7; co-cultures: *n* = 3; mean ± standard error of the mean.

To determine the effect of different culture media and/or co-culture with HUVEC on the functionality of hiPSC-derived HLC, the activity of different pharmacologically relevant CYP isoenzymes was investigated by analyzing isoenzyme-specific product formation rates after application of a substrate cocktail (Figure 8). All measured CYP activities were clearly higher in HCM-I/II + EGM complete or in HCM-I/II + EGM supplements maintained with or without HUVEC as compared with HCM-I/II control cultures. Only for CYP1A2 differentiation with HCM-I/II + EGM complete in co-culture with HUVEC resulted in product formation rates similar to the HCM-I/II control (Figure 8A). And differentiation using HCM-I/II + EGM supplements maintained with or without HUVEC showed higher activities than HCM-I/II + EGM complete. CYP2B6 showed significantly higher activities for HCM-I/II + EGM complete with and without HUVEC and for HCM-I/II + EGM supplements when compared with HCM-I/II control (*p* < 0.05; Figure 8B). Activity patterns for CYP3A4 were similar showing significantly higher activities for both co-cultures and for mono-cultures using HCM-I/II + EGM supplements (*p* < 0.05; Figure 8C).

## 3. Discussion

To date, the use of hiPSC-derived HLC in pharmacological drug screening and toxicity testing is limited by their low hepatic functionality due to a heterogeneous phenotype of HLC [26] resembling rather fetal than adult primary human hepatocytes [11]. To improve the differentiation outcome of hiPSC/hESC, several approaches have been focusing on co-culture with non-parenchymal cell types during the differentiation process, using various cell types and culture systems. A major precondition to a functional co-culture system is the use of a suitable culture medium, which meets the requirements of hiPSC as well as those of co-cultured cell types. In the present study, the influence of different culture medium compositions on HUVEC and hiPSC-derived DE cells maintained separately or in co-culture with each other was investigated. Culture media tested included media in use for hiPSC differentiation (HCM), media for HUVEC culture (ECM) and different mixtures of both.

The results from testing different media compositions in HUVEC mono-cultures showed that HUVEC did not survive using pure HCM-I, whereas use of HCM-I + EGM complete resulted in a similar growth behavior as EGM complete, and use of HCM-I + EGM supplements also supported HUVEC growth, although at a reduced level. These findings are in accordance with studies by Takebe et al. [25], who successfully employed HCM and EGM (both from Lonza) for co-culture of hiPSC with HUVEC and mesenchymal stem cells. The observation that HUVEC did not grow in HCM-I can be explained by the fact that this medium lacks some of the ingredients of EGM complete (Table S3), e.g., bovine hypothalamic extract containing the potent endothelial mitogen endothelial cell growth factor (ECGF) [27]. ECGF is even more efficient in combination with heparin [28] also being part of EGM complete, but not being present in HCM. Furthermore, HCM does not contain basic fibroblast growth factor (bFGF), which binds to heparin leading to dimerisation of bFGF receptors [29] and selective induction of endothelial cell proliferation [30]. Insulin, which is included in HCM, but not in EGM complete, is also reported to increase mitosis in endothelial cells [31], but this effect maybe counteracted by other factors. For example, transferrin, which is also part of HCM, was shown to have no influence on endothelial cell proliferation [32], but may play a role in promoting oxidant-induced apoptosis [33]. Furthermore, HCM contains ascorbic acid, which was reported to anticipate oxidative stress-induced apoptosis in endothelial cells [34]. In addition, the reduced proliferation of HUVEC cultivated in HCM-I + EGM supplements compared with cultivation in EGM complete might be associated with the higher glucose concentration of HCM-I + EGM supplements medium (10 mM), as high glucose concentrations have been shown to increase apoptosis and oxidative stress in endothelial cells [35]. In particular constant exposure to glucose levels above 7 mM, which is defined as hyperglycemia in the blood, is not physiological and may harm the endothelium [36].

Based on the results from media testing in HUVEC mono-cultures both, HCM-I/II + EGM complete and HCM-I/II + EGM supplements were tested for their suitability to induce hepatic differentiation in hiPSC-derived DE cells in presence or absence of HUVEC. The results were compared with those from using pure HCM-I/II.

As indicated by stage-specific marker expression and CYP activities, both test media improved the hepatic differentiation of hiPSC as compared with pure HCM-I/II, regardless whether HUVEC were present or not. The favorable effects of EGM complete or EGM supplements on hepatic differentiation of hiPSC may be due to some of the factors contained in those media (Table S3). In particular, the growth factor bFGF contained in EGM supplements has been shown to support the differentiation of DE cells into hepatoblasts in a concentration-dependent manner [37] and has been employed in some studies [17,38]. This may explain the distinctly increased gene expression of the hepatoblast marker *AFP* in cultures treated with HCM-I/II + EGM supplements, since this medium contains the highest amount of bFGF. The secretion of AFP was significantly higher in all test groups as compared with the control cultures maintained in pure HCM-I/II. A higher grade of hepatoblast differentiation may consequently lead to an increased hepatic maturation, as indicated by distinctly increased albumin secretion rates in all test groups. The albumin secretion detected in the test groups, when calculated for the same time interval (days), was more than twice as high compared to other studies [9,16,39,40], while Baxter et al. and Gieseck et al. reported even higher albumin secretion rates of up to 1.5 g/day/10^6^ cells [11,22]. However, it has to be considered that the authors of these studies have been using different cell lines and culture protocols, which might have influenced the differentiation outcome and albumin secretion.

Potential reasons for the observed variability in the present data sets can be seen in influencing factors showing some variations among the experiments, e.g., passage number and seeding efficiency of different cell batches. In addition the usage of different batches of medium compounds may cause some variances. For example, B27 supplement used for the definitive endodermal differentiation step showed substantial variation between specific lots of B27 supplements in neuronal cell cultures [41] and also in hepatic differentiation experiments [42].

The gene expression of the epithelial marker *KRT18* was only slightly increased in differentiated HLC compared with undifferentiated hiPSC; however, immunoreactivity for KRT18 was clearly observed in hiPSC-derived HLC cultures. Both, gene and protein expression of KRT18, were barely influenced by the medium composition or addition of HUVEC. In contrast, the gene expression of the hepatic transcription factor *HNF4A* was distinctly increased in all groups compared with the use of pure HCM-I/II. This observation might again be explained by the effect of bFGF contained in HCM-I/II + EGM complete and HCM-I/II + EGM supplements since gene expression of *HNF4A* was shown to be induced by BMP4/bFGF supplemented media [10,43]. In addition, there are data indicating that the expression of *HNF4A* can be influenced by exposure to glucocorticoids [44], which are contained at a higher level in both test media as compared with pure HCM-I/II. Interestingly there was a discrepancy between protein expression of HNF4A as analyzed by immune fluorescence staining and gene expression of that factor. This could be explained by post-translational modifications of the protein that may reduce the sensitivity of the antibody used for immune fluorescence staining. Yokoyama et al. reported eight different post-translational modifications sites and observed that one of these sites is even changing in response to varying glucose levels [45], as occurring in different culture medium compositions used in this study. The nuclear receptor HNF4A is also responsible for the transcriptional activation of several CYP isoenzymes such as CYP1A2 [46] and CYP3A4 [47]. Both, the significantly increased *HNF4A* expression and the higher hydrocortisone concentration in both test media can explain the significant increase in CYP activities observed in the present study. Glucocorticoids are known to induce CYP2B, CYP2C and CYP3A in humans [48]. CYP activities were significantly increased in cultures differentiated in the optimized co-culture media as compared to the HCM-I/II control group. Maximal activities reached up to 10% of the activities of primary human hepatocytes cultured for 24 h, which were determined in a previous study of the authors [23].

The presence of HUVEC in hiPSC co-cultures could be confirmed by light-microscopy. In addition, HUVEC were detected at the end of hepatic differentiation by gene and protein expression of the endothelial cell marker PECAM1. It was reported that heparin, which is included in EGM supplements, enhances HGF production at a post-transcriptional level in HUVEC [49]. This may promote hepatic maturation [50] and proliferation [10,51] in co-cultures. However, in the present study, no supportive effect of HUVEC on the hepatic differentiation of hiPSC was observed.

An overview of current in vitro co-culture approaches for hepatic differentiation of human pluripotent stem cells is provided in Table 1. So far, the use of HUVEC to support hepatic differentiation of hiPSC was reported only in co-cultures together with either human mesenchymal stem cells [25] or adipose derived stem cells [52]. Takebe and colleagues created highly functional liver buds, which were able to rescue drug-induced lethal liver failure in immunodeficient mice. Additionally they observed that culturing hiPSC-derived HLC with endothelial cells alone failed to form three-dimensional transplantable tissues. Ma and coworkers observed significantly increased *ALB*, *HNF4A* and transthyretin gene expression in their co-culture model compared with hiPSC mono-cultures [52]. Interestingly, in contrast to the present study, both studies [25,52] omitted the epidermal growth factor (EGF) in the HCM used for differentiation. Since EGF was shown to stimulate cell proliferation in HUVEC in a dose-dependent manner [53,54], EGF should not have a negative effect on HUVEC during co-culture experiments. Another study applied hiPSC-derived endothelial cells for co-culture during hepatic differentiation of hiPSC and was able to show significantly increased albumin secretion [55].

To increase the effect of HUVEC co-culture on hepatic maturation of hiPSC in the here described model, influencing factors such as the number of HUVEC in relation to hiPSC-derived DE cells, and the culture technique should be optimized. For example, Transwells [56] or niches separated by different extracellular matrices [55,57] can be used to provide a larger growth area for HUVEC, in a separate compartment from hiPSC (Table 1). Another approach would be the detachment of hiPSC-derived DE cells, which can then be mixed with HUVEC and reseeded [25,58,59]. These approaches would also enable to add the HUVEC at a later stage of differentiation, namely the hepatic endoderm stage as described in vitro by Takebe et al. [25] and in vivo by Matsumoto et al. [24]. Since in the present study HUVEC were expected to adhere in free spaces between the DE cells, the co-culture was initiated before spreading of DE cells resulting in a decrease of the available adhesion area for HUVEC.

As becomes apparent in Table 1, the most frequently used cell type in current co-culture approaches for hepatic differentiation of human pluripotent stem cells are murine embryonic fibroblasts [57,59,60,61], which increased hepatic gene expression and functionality. In the present study HUVEC were chosen as a well-established and standardized cell source for parenchymal-endothelial cell co-cultures. Furthermore, the umbilical vein is the major afferent vessel in the fetal liver [62,63] and HUVEC might thereby be important for the embryonic liver development. In this context, HUVEC were already successfully applied as early supporters of hepatic differentiation in previous studies [25,52]. Another interesting approach would be the usage of tissue-specific endothelial cells for support of hepatic differentiation through the secretion of tissue-specific factors. It was shown for several organs that tissue-specific endothelial cells orchestrate organ development as well as regeneration after injury before building a functional vasculature [64]. Ding et al. could show that liver sinusoidal endothelial cells release factors, which initiate and sustain liver regeneration induced by partial hepatectomy in mice [65]. Furthermore, there is evidence that adult hepatocytes also play a role in stem cell fate decision during liver regeneration by releasing growth factors such as HGF, Wnt and FGFs [66]. Hence, another strategy would be the co-cultivation with primary adult hepatocytes during hepatic differentiation.

In future studies the present findings should be verified using additional hiPSC lines to identify a potential dependency on donor-specific and epigenetic characteristics of individual hiPSC lines. In addition, a closer investigation of individual factors and compounds in the culture media mixtures would be helpful to create well-defined culture media formulations and to facilitate the further improvement of co-culture media.

## 4. Materials and Methods

### 4.1. Culture of HUVEC

Cryopreserved HUVEC (PromoCell GmbH, Heidelberg, Germany) were thawed as recommended by the manufacturer and were cultivated in endothelial cell growth medium (PromoCell GmbH), consisting of basal medium and supplements (EGM complete) and 0.05 mg/mL gentamycin (Merck, Darmstadt, Germany) on cell culture dishes (ThermoScientific, Waltham, MA, USA) at 37 °C in a 5% CO_2_ atmosphere. The cells were passaged according to the manufacturer’s instructions when they reached around 95% confluence. The composition of EGM complete as provided by the manufacturer is shown in Table S3.

### 4.2. Culture and Hepatic Differentiation of hiPSC

The hiPSC line DF6-9-9T [68] (WiCell Research Institute, Madison, WI, USA) was cultured under feeder-free conditions on Nunclon^TM^ six-well cell culture plates (ThermoScientific NuncTM, Schwerte, Germany) coated with 8.68 µg/cm² Matrigel (growth factor reduced, Corning, NY, USA). Cells were expanded with mTeSR^TM^1 medium (Stemcell Technologies, Vancouver, BC, Canada) containing 0.05 mg/mL gentamycin (Merck, Darmstadt, Germany).

Hepatic differentiation of hiPSC was performed according to protocols from Hay et al. [15,18,69] with some modifications, as described previously [23]. Briefly, when hiPSC reached a confluence of approximately 70%, differentiation into DE cells was induced with Roswell Park Memorial Institute (RPMI) 1640 culture medium (Merck) supplemented with 100 ng/mL activin A (Peprotech, London, UK), 50 ng/mL Wnt3a (R&D Systems, Minneapolis, MN, USA), 1 µM sodium butyrate (Sigma-Aldrich, St. Louis, MO, USA) and 2% (*v*/*v*) B27 supplements without insulin (Life Technologies, Carlsbad, CA, USA) for three days. Subsequently DE cells were differentiated into hepatoblasts over 13 days with hepatocyte culture medium consisting of basal medium and single quots (Lonza, Walkersville, MD, USA) and 10 ng/mL HGF (Peprotech, Rocky Hill, NJ, USA). For further maturation to hepatocyte-like cells 10 ng/mL OSM (Peprotech) were added during the last four days of differentiation. The hepatoblast differentiation medium is referred to as HCM-I and the maturation medium is referred to as HCM-II throughout the whole manuscript. The composition of HCM as provided by the manufacturer is shown in Table S3.

### 4.3. Culture Medium Testing in Mono-Cultures of HUVEC

For testing of different culture media, HUVEC were seeded at a density of 4 × 10^3^ cells/cm^2^ and cultured over 14 days in the presence of 100% EGM complete (positive control), 100% HCM-I, HCM-I and EGM complete at a ratio of 1:1 (HCM-I + EGM complete) or HCM-I enriched with endothelial cell growth supplements (HCM-I + EGM supplements).

### 4.4. Co-Culture of hiPSC-Derived DE cells with HUVEC

For co-culture experiments, hiPSC were differentiated into DE cells as described above. Further differentiation was carried out in HCM-I/II + EGM complete or in HCM-I/II + EGM supplements with HUVEC added to the DE cells at a ratio of 1:2 (5 × 10^5^ HUVEC + 1 × 10^6^ DE cells). In parallel, DE cells were differentiated in HCM-I/II + EGM complete or in HCM-I/II + EGM supplements without HUVEC. An overview of culture media and culture medium combinations used for testing in HUVEC or hiPSC cultures is provided in Table 2.

### 4.5. Analyses of Biochemical Parameters

The metabolic activity of HUVEC was assessed by daily measurement of glucose and lactate concentrations with a blood gas analyzer (ABL 700, Radiometer, Copenhagen, Denmark). Potential cell damage was detected by analyzing the release of LDH using an automated clinical chemistry analyzer (Cobas^®^ 8000; Roche Diagnostics, Mannheim, Germany). The secretion of the albumin precursor protein AFP and urea during hiPSC differentiation was detected also using an automated clinical chemistry analyzer (Cobas^®^ 8000, Roche Diagnostics). Albumin secretion, as a marker for mature hepatocytes, was quantified using an ELISA Quantitation kit and 3′,5,5′-tetramethylbenzidine substrate (both from Bethyl Laboratories, Montgomery, TX, USA) according to the manufacturer´s instructions.

### 4.6. Gene Expression Analysis

RNA was isolated from undifferentiated hiPSC, HLC, HUVEC, or HLC-HUVEC co-cultures. Isolation of RNA and subsequent cDNA synthesis were performed as described elsewhere [70], using PureLink^TM^ RNA Mini Kit (Life Technologies) and High Capacity cDNA Reverse Transcription Kit (Applied Biosystems, Foster City, CA). Each cDNA template was mixed with PCR Master mix (Applied Biosystems) and human-specific primers and probes (TaqMan GeneExpression Assay system, Life Technologies, Table 3). Quantitative real-time PCR (qRT-PCR) was performed using a Realtime cycler (Mastercycler ep Realplex 2, Eppendorf, Hamburg, Germany). The expression of specific genes was normalized to that of the housekeeping gene glyceraldehyde-3-phosphate dehydrogenase (*GAPDH*) and fold changes of expression levels were calculated with the ΔΔ*C*t method [71].

### 4.7. Immunocytochemical Staining

Immunofluorescence staining was performed as described elsewhere [70]. Antibodies used are listed in Table 4. Staining of hiPSC-derived cultures was performed in 24-well plates (lumox®, Sarstedt, Nümbrecht-Rommelsdorf, Germany), while HUVEC were cultured and subsequently fixed on chamber slides (Thermo Scientific™ Nunc™ Lab-Tek™ II Chamber Slide™ System) for immunocytochemical analysis.

Fluorescence microscopic pictures were analysed by means of the open source image processing program ImageJ recording at least 5 visual fields for each group.

### 4.8. Measurement of Cytochrome P450 (CYP) Isoenzyme Activities

Activities of the pharmacologically relevant CYP isoenzymes CYP1A2, CYP2B6 and CYP3A4 were measured in hiPSC after completion of hepatic differentiation as described previously [23]. Briefly, a cocktail containing the CYP substrates phenacetin (CYP1A2), bupropion (CYP2B6) and midazolam (CYP3A4) was added to the cultures and the formation of the corresponding isoenzyme specific products was analyzed by LC-MS as described previously [23].

### 4.9. Statistical Evaluation

Experiments were performed in three to eight repeats, as indicated in the figure legends, and results are presented as mean ± standard error of the mean. The area under the curve was calculated for time-courses of biochemical parameters and differences between culture media and/or co-cultures were detected with a subsequent unpaired, two-tailed Student´s *t*-test. Differences were judged as significant, if the *p*-value was less than 0.05.

## 5. Conclusions

In summary, the application of co-cultures to generate functional hiPSC-derived HLC is a relatively new and complex research field. Our study shows that the establishment of a functional co-culture model requires an intense study of surrounding aspects influencing the cell maintenance. Particular attention should be given to the composition of the applied media, as our results show that the effect of the co-culture medium outweighed the effect of the co-culture itself.

## Figures and Tables

**Table 1:** Studies on hepatic differentiation of human induced pluripotent stem cells (hiPSC) or human embryonic stem cells (hESC) in co-culture with different cell types.

Cell Type Used for Co-Culture with hiPSC/hESC	Ratio (hiPSC/hESC-Derived Cells:Co-Cultured Cell Type(s))	Co-Culture Method	Culture Medium Applied	Ref.
Murine hepatic stromal cell line MLSgt20	1:1	Self-aggregation in microwells	DMEM + FBS + differentiation factors	[58]
Murine embryonic fibroblasts (3T3-J2)	0.04:1	Seeding of DE cells onto mitomycin-treated 3T3-J2 feeder cells	Hepatocyte culture medium + FBS	[60]
Murine embryonic fibroblasts (swiss 3T3)	Not specified	Hepatoblast monolayer covered with 3T3 cell sheet	L15 medium + differentiation factors	[61]
HUVEC and mesenchymal stem cells	10:7:2	Spontaneous formation of 3D liver buds	HCM (without EGF) + EGM, 1:1	[25,67]
hiPSC-derived endothelial cells	2 : 1	Multicomponent hydrogel fibers containing galactose for HLC and collagen for endothelial cells	Not specified	[55]
Hepatic stellate cell line TWNT-1	Not specified	Cell inserts	DMEM-F12 + knockout serum replacer + DMSO	[56]
Murine embryonic fibroblasts (3T3-J2)	2:1 or 2.5:1 for cryopreserved HLC	Micropatterned co-culture containing collagen coating and matrigel overlay	RPMI + B27 supplement + differentiation factors	[57]
Murine embryonic fibroblasts (3T3-J2)	2:1	Self-aggregation in microwells	RPMI + B27 supplement + differentiation factors	[59]
Primary rat hepatocytes	1:2	Microfluidic co-culture	DMEM + FBS + maintenance factors and IMDM + FBS + DMSO + differentiation factors	[66]
HUVEC and adipose derived stem cells	1:1:0.02	3D bioprinting of in vivo like liver lobule structures	HCM (without EGF) + EGM-2, 1:1	[52]
HUVEC	2:1	HUVEC grow in free spaces of hiPSC-derived DE cell monolayers	HCM + EGM complete, 1:1 and HCM + EGM Supplements	Present study

**Table 2:** Culture media and culture medium combinations used for testing in cultures of human umbilical vein endothelial cells (HUVEC) or human induced pluripotent stem cells (hiPSC).

Component	EGM complete	HCM-I	HCM-II	HCM-I/II + EGM Complete	HCM-I/II + EGM Supplements
Hepatocyte culture medium (HCM) Bullet Kit	-	100% (*v*/*v*)		50% (*v*/*v*)	97.5% (*v*/*v*)
Endothelial cell growth medium (EGM)	97.5% (*v*/*v*)	-		48.75% (*v*/*v*)	-
EGM Supplements	2.5% (*v*/*v*)	-		1.25% (*v*/*v*)	2.5% (*v*/*v*)
Human hepatocyte growth factor, recombinant	-	10 ng/mL	10 ng/mL	10 ng/mL	10 ng/mL
Human oncostatin M, recombinant	-	-	10 ng/mL	10 ng/mL ^1^	10 ng/mL ^1^
Gentamycin	0.05 mg/mL	0.05 mg/mL	0.05 mg/mL	0.05 mg/mL	0.05 mg/mL

^1^ only added to media containing HCM-II.

**Table 3:** Applied Biosystems TaqMan Gene Expression Assays^®^.

Gene Symbol	Gene Name	Assay ID
*AFP*	α fetoprotein	HS00173490_m1
*ALB*	albumin	HS00910225_m1
*GAPDH*	glyceraldehyde-3-phosphate dehydrogenase	HS03929097_g1
*HNF4A*	Hepatocyte nuclear factor 4, α	Hs00230853_m1
*KRT18*	Keratin 18	Hs02827483_g1
*PECAM1*	platelet and endothelial cell adhesion molecule 1	Hs00169777_m1
*POU5F1*	POU domain, class 5, transcription factor 1	HS00999632_g1
*VWF*	von Willebrand factor	Hs00169795_m1

**Table 4:** Primary and secondary antibodies used for immunofluorescence staining.

Antibody Type and Specifity	Protein Symbol	Species	Manufacturer	Article-No.	Final Conc. (µg/mL)
Primary Antibody					
Cytokeratin 18	CK18	mouse	Santa Cruz	Sc-6259	2
Hepatocyte nuclear factor 4 α	HNF4A	rabbit	Santa Cruz	Sc-8987	4
Platelet endothelial cell adhesion molecule 1	PECAM1	mouse	Abcam	ab24590	5
POU domain, class 5, transcription factor 1	OCT3	rabbit	Santa Cruz	Sc-9081	2
Von Willebrand factor	VWF	rabbit	Abcam	ab6994	35.5
Secondary antibody					
Alexa Fluor® 488 anti-mouse		goat	Life Technologies	A-11029	2
Alexa Fluor® 594 anti-rabbit		goat	Life Technologies	A-11037	2

## Supplementary Materials

Supplementary materials can be found at www.mdpi.com/1422-0067/18/8/1724/s1.

## Abbreviations

AFP	α-fetoprotein
ALB	Albumin
bFGF	Basic fibroblast growth factor
BMP	Bone morphogenetic proteins
CYP	Cytochrome P450
DE	Definitive endoderm
DMEM	Dulbecco’s modified eagle’s medium
DMSO	Dimethyl sulfoxide
ECGF	Endothelial cell growth factor
EGF	Epidermal growth factor
EGM	Endothelial cell growth medium
FBS	Fetal bovine serum
FGF	Fibroblast growth factor
GAPDH	Glyceraldehyde-3-phosphate dehydrogenase
HCM	Hepatocyte culture medium
hESC	Human embryonic stem cells
HGF	hepatocyte growth factor
hiPSC	Human induced pluripotent stem cells
HLC	Hepatocyte-like cells
HNF4A	Hepatocyte nuclear factor 4 α
HUVEC	Human umbilical vein endothelial cells
KRT18	Cytokeratin 18
L15	Leibovitz’s
LDH	Lactate dehydrogenase
OSM	Oncostatin M
PECAM1	Platelet and endothelial cell adhesion molecule 1
POU5F1	POU domain, class 5, transcription factor 1
RPMI	Roswell Park Memorial Institute
VWF	Von Willebrand factor
