# Supplementary material for: Effects of Co-Culture Media on Hepatic Differentiation of hiPSC with or without HUVEC Co-Culture
Source: Int J Mol Sci. 2017 Aug 7;18(8):1724. doi: 10.3390/ijms18081724 (PMC5578114; doi:10.3390/ijms18081724)
Supplement: Supplementary file 1 [file ijms-18-01724-s001.pdf]

Supplementary Table S1. Percentages of cells positive for stage-specific markers in mono-cultures of human umbilical vein endothelial cells (HUVEC), mean ± standard deviation. n.d. = not detected

	PECAM1	HNF4A	KRT18	VWF
**EGM complete**	91 ± 6	n.d.	n.d.	79 ± 5
**HCM-I + EGM complete**	71 ± 23	n.d.	n.d.	83 ± 14
**HCM-I + EGM Supplements**	73 ± 13	n.d.	n.d.	62 ± 12

Supplementary Table S2. Percentages of cells positive for stage-specific markers in cultures of human induced pluripotent stem cells (hiPSC), mean ± standard deviation. n.d. = not detected

	POU5F1	KRT18	HNF4A	PECAM1
**Undiff. hiPSC**	99 ± 3	n.d.	n.d.	n.d.
**HCM-I/II**	n.d.	80 ± 6	60 ± 30	n.d.
**HCM-I + EGM complete**	n.d.	47 ± 14	28 ± 19	n.d.
**HCM-I + EGM supplements**	n.d.	49 ± 14	20 ± 15	n.d.
**HCM-I + EGM complete +HUVEC**	n.d.	60 ± 17	11 ± 6	37 ± 17
**HCM-I + EGM supplements + HUVEC**	n.d.	38 ± 10	16 ± 15	23 ± 26

Supplementary Table 3. Compositions of basal media as provided by the manufacturers

Component	HCM (Lonza)	EGM complete (PromoCell)
Glucose	10 mM	5.6 mM
FCS / BSA	BSA: fatty acid free [1]	FCS: 0.02 ml/ml
Bovine hypothalamic extract (rich in endothelial cell growth factor)	/	0.004 ml/ml
Epidermal Growth Factor (recombinant human)	Included [1]	0.1 ng/ml
Basic Fibroblast growth factor (recombinant human)	/	1 ng/ml
Heparin	/	90 µg/ml
Hydrocortisone	Included [1]	1µg/ml
Insulin	Included [1]	/
Transferrin	Included [1]	/
Ascorbic acid	Included [1]	/

[1] Concentrations of supplements not published
